# Endocrine and Metabolic Characteristics and Prognosis of Long COVID Following SARS-CoV-2 Infection During the Omicron Period

**DOI:** 10.3390/jcm15218492

**Published:** 2026-11-01

**Authors:** Daisuke Omura, Yoshiaki Soejima, Yuki Otsuka, Yasuhiro Nakano, Kazuki Tokumasu, Yui Matsuda, Yasue Sakurada, Ryosuke Takase, Atsuhito Suyama, Marina Kawaguchi, Toru Hasegawa, Hiroyuki Honda, Keigo Ueda, Fumio Otsuka

**Affiliations:** Department of General Medicine, Graduate School of Medicine, Dentistry and Pharmaceutical Sciences, Okayama University, Okayama 700-8558, Japan; p32v0ja8@s.okayama-u.ac.jp (Y.S.); otsuka@s.okayama-u.ac.jp (Y.O.); y-nakano@okayama-u.ac.jp (Y.N.); tokumasu@okayama-u.ac.jp (K.T.); phvw0350@okayama-u.ac.jp (Y.M.); pzaf6h9w@s.okayama-u.ac.jp (Y.S.); p4v05asb@okayama-u.ac.jp (R.T.); asuyama@s.okayama-u.ac.jp (A.S.); p1ka7qnp@s.okayama-u.ac.jp (M.K.); pwcp0od9@s.okayama-u.ac.jp (T.H.); ppgf1hrd@s.okayama-u.ac.jp (H.H.); p02n620b@okayama-u.ac.jp (K.U.)

**Keywords:** body mass index, long COVID, obesity, omicron variant, post-COVID-19 condition

## Abstract

**Background:** The clinical features and recovery of patients with obesity and Long COVID remain incompletely understood. **Methods:** We retrospectively analyzed 903 patients attending a COVID-19 aftercare clinic during the Omicron period. Biomarkers were compared across body mass index (BMI) categories. Recovery was defined as physician-determined conclusion of outpatient follow-up following sufficient symptom improvement, not necessarily complete resolution. Recovery analyses were exploratory; 572 patients comprised a sensitivity cohort clinically compatible with the World Health Organization (WHO) post-COVID-19 condition definition. **Results:** Serum ferritin levels were higher in overweight and obese men than in normal-weight men. Cortisol/adrenocorticotropic hormone (ACTH) ratios in women and free thyroxine (FT4) levels in men differed across BMI categories. Recovery analysis from symptom onset included 565 participants and 186 events. Men with obesity had a lower recovery hazard than normal-weight men, with consistent direction across sensitivity analyses (age-adjusted pooled model, *n* = 565; hazard ratio: 0.390; 95% CI: 0.170–0.894), based on six recovery events among men with obesity. The main sex-by-obesity interaction was nonsignificant; additional interaction results varied by model. The WHO-compatible recovery analysis was limited by sparse events. **Conclusions:** Obesity was associated with a longer time to clinically defined recovery among men, alongside BMI-related endocrine–metabolic differences. These exploratory findings warrant confirmation; sparse events and residual confounding preclude causal or definitive sex-specific conclusions.

## 1. Introduction

Long COVID, also referred to as post-COVID-19 condition or post-acute sequelae of SARS-CoV-2 infection (PASC), is a heterogeneous syndrome in which symptoms persist, relapse, or emerge after acute SARS-CoV-2 infection. The World Health Organization defines the condition in people with a history of probable or confirmed SARS-CoV-2 infection as symptoms present usually around three months after illness onset, lasting at least two months and not explained by another diagnosis [1]. Common manifestations include fatigue, post-exertional symptom exacerbation, cognitive dysfunction, dyspnea, palpitations, sleep disturbance, pain, gastrointestinal symptoms, and autonomic complaints [2,3]. Large cohort and survey studies have shown that Long COVID may affect multiple organ systems and impair daily function, social participation, and work capacity [2,3,4,5].

Several factors have been associated with Long COVID, including female sex, older age, high acute symptom burden, pre-existing comorbidities, severe acute infection, type 2 diabetes, and higher body mass index (BMI) [4,6,7,8,9]. Obesity is of interest because it is associated with severe acute COVID-19 and may indicate inflammatory, cardiometabolic, and sleep-related vulnerability [8,10,11,12,13,14]. These factors overlap with proposed Long COVID mechanisms, including persistent immune activation, endothelial injury, microvascular dysfunction, viral persistence, autoimmunity, and autonomic dysregulation [3,15,16,17,18,19,20,21,22,23]. However, BMI has often been treated as a risk factor or adjustment variable rather than as a clinical context for biomarker heterogeneity among patients who already have Long COVID.

Endocrine and metabolic abnormalities may also be relevant to Long COVID. Previous studies have reported cortisol-related, adrenal, pituitary, thyroid, and broader endocrine findings after COVID-19, but results have been heterogeneous across cohorts and methods [19,24,25,26,27,28,29,30]. Recent dynamic stimulation testing in a highly selected cohort of 16 patients with refractory Long COVID found largely preserved peak anterior pituitary responses but delayed response kinetics, illustrating that isolated basal hormone measurements cannot characterize dynamic hypothalamic–pituitary responses [31]. Obesity also modifies endocrine physiology: circulating cortisol may be normal or low despite increased tissue-level glucocorticoid action through 11β-hydroxysteroid dehydrogenase type 1, and obesity is often associated with mild TSH elevation through leptin-mediated hypothalamic stimulation and altered thyroid hormone metabolism [32,33,34,35,36,37,38]. Underweight status may reflect a different biological context, including lower physiologic reserve, appetite loss, gastrointestinal symptoms, catabolic stress, autonomic dysfunction, and selective nutritional vulnerability.

Metabolic–inflammatory and nutrition-related markers may provide context for comparing clinical laboratory profiles across BMI categories. Ferritin is an iron-storage protein and acute-phase reactant that can be influenced by inflammation, adiposity, hepatic metabolism, infection history, and comorbid conditions. Trace elements and nutrition-related markers, including zinc, folate, vitamin B1, and cholinesterase, may also be affected by dietary intake, inflammation, hepatic metabolic status, and supplement or prescription use [13,39,40,41,42,43]. These variables are clinically relevant in Long COVID [44], where fatigue, appetite loss, gastrointestinal symptoms, and supplement use are frequently encountered, but they require cautious interpretation in retrospective clinical datasets. In a phase-stratified study, we previously reported a difference in ferritin levels between women with myalgic encephalomyelitis/chronic fatigue syndrome (ME/CFS) and those without ME/CFS in an earlier COVID-19 period, with a more modest difference in the Delta- and Omicron-dominant periods [45].

The primary objective of this exploratory retrospective study was to characterize clinical, endocrine, and metabolic features across BMI categories among patients with persistent post-COVID-19 symptoms, with particular interest in obesity. As a secondary exploratory outcome, we also examined the association between obesity and the time from symptom onset to clinically defined recovery, marked by the conclusion of outpatient follow-up at the COVID-19 aftercare (CAC) clinic. This research concerned differences within a symptomatic clinic cohort, rather than the population risk of developing Long COVID. Sex-stratified results and interaction analyses were exploratory.

## 2. Patients and Methods

### 2.1. Study Design and Inclusion of Patients with Long COVID

**Figure 1:**
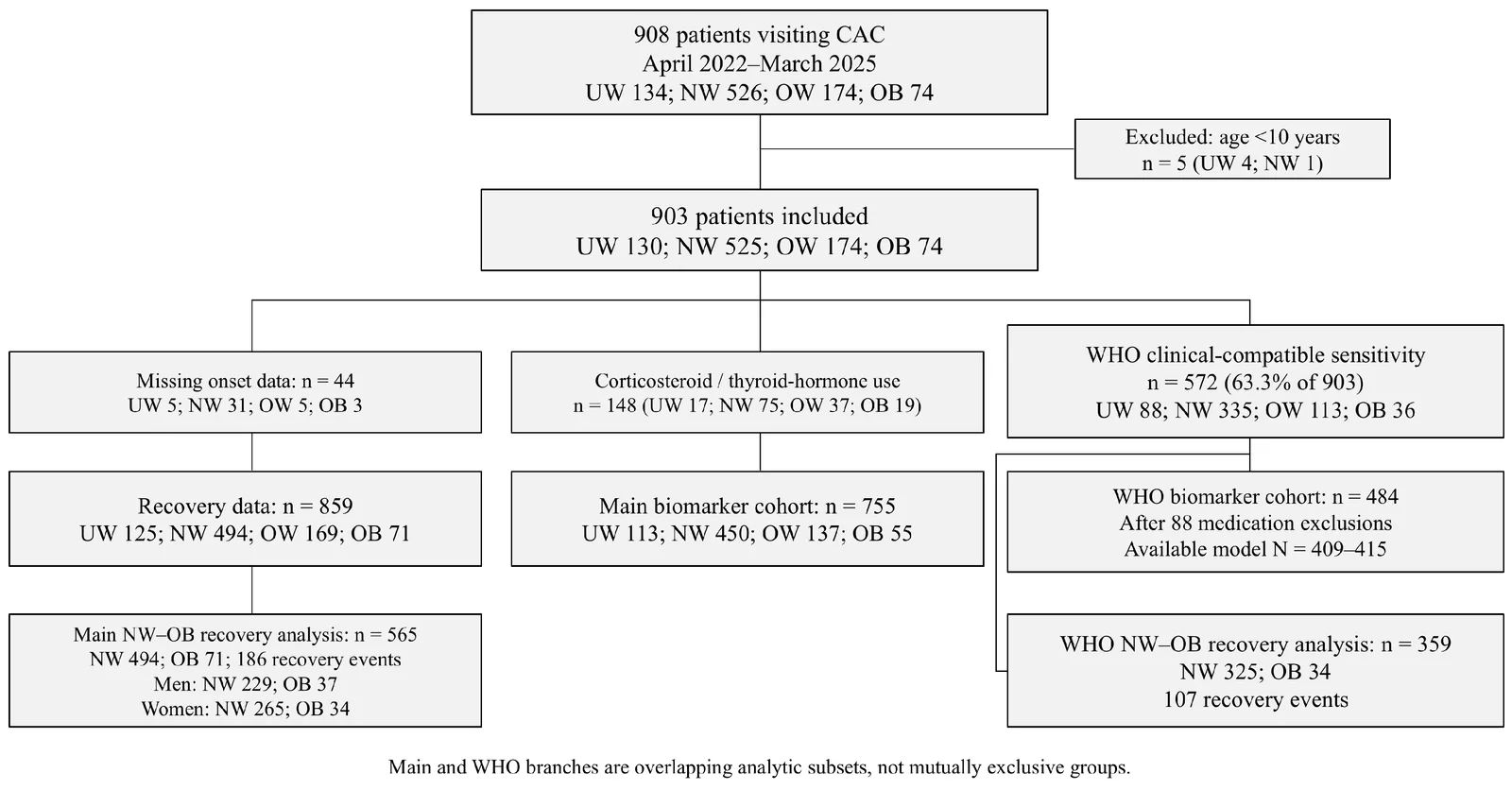
**Participant selection and overlapping analytic cohorts.** The WHO-compatible sensitivity cohort is nested within the main clinic cohort; the two branches should not be summed. Corticosteroid or thyroid hormone exclusions apply only to biomarker analyses. The recovery analyses compare NW and OB participants without these medication exclusions. Marker-specific sample sizes are reported with the relevant analyses. BMI-stratified groups: UW, <18.5 kg/m^2^; NW, 18.5–24.9 kg/m^2^; OW, 25.0–29.9 kg/m^2^; OB, ≥30.0 kg/m^2^ (UW, underweight; NW, normal weight; OW, overweight; OB, obesity). CAC, COVID-19 aftercare.

This retrospective cohort study included patients evaluated at the dedicated COVID-19 aftercare (CAC) clinic at Okayama University Hospital, Okayama, Japan. The study included first visits to the CAC clinic from 1 April 2022, to 31 March 2025, which was treated as an Omicron-era calendar period; infecting variants and subvariants were not individually confirmed for all participants. Patients were referred for persistent or relapsing symptoms after confirmed or suspected SARS-CoV-2 infection; the operational definition of Long COVID for this clinic cohort was these symptoms lasting at least one month and not being explained by an alternative diagnosis. This early operational threshold supported clinic referral and study inclusion but was not treated as equivalent to the WHO post-COVID-19 condition definition. For the WHO clinical-compatibility sensitivity analysis, longitudinal records had to support symptoms at approximately three months after onset or later with a duration of at least two months; persistence, fluctuation, and relapse were considered when demonstrated by the recorded trajectory. The cohort was selected based on the clinical judgment of the outpatient physicians who assessed and followed the patients, together with the available longitudinal records. Their clinical assessments and reassessments supported attribution to post-COVID-19 illness rather than another diagnosis. Absence of a stored positive PCR or antigen result for the index episode was not, by itself, a reason for exclusion, consistently with the WHO allowance for probable as well as confirmed infection. The resulting 572-person sensitivity cohort was considered clinically compatible with the WHO PCC definition based on the available longitudinal records and the original clinical case ascertainment, incorporating clinical reassessment. This was a nested subset of the main 903-person clinic cohort, used to assess whether findings depended on the broader clinic-based inclusion criterion (Figure 1).

The analytic cohort comprised 903 participants. For biomarker analyses, 148 participants (16.4%) were excluded because they received corticosteroids, thyroid hormone replacement, or both. These treatments can directly alter adrenocortical or thyroid measurements. The included and excluded groups had median ages of 41 and 44 years; 51.5% and 56.8% were female, and the median BMI values were 22.1 and 23.0 kg/m^2^. Missing laboratory values were excluded only from the corresponding marker-specific analysis. Recovery was defined as the termination of visits to the CAC clinic once symptoms had sufficiently improved and the attending physician determined that continued follow-up was no longer necessary. Complete symptom resolution was not required. This definition is consistent with published methodology from the same CAC clinic setting [46] but is not a validated symptom or functional scale. Patients still under follow-up without recovery were censored on 31 March 2026, and those lost to follow-up or referred elsewhere were censored at the last known contact; other reasons for ending follow-up were not classified as recovery. Time to recovery was measured from symptom onset to recovery or censoring. Additional analyses accounting for the first visit to the CAC clinic are described in Section 2.5.

### 2.2. Categorization of Patients with Long COVID by BMI

BMI was calculated as weight in kilograms divided by height in meters squared. Participants were categorized as UW (<18.5 kg/m^2^), NW (18.5–24.9 kg/m^2^), OW (25.0–29.9 kg/m^2^), or OB (≥30.0 kg/m^2^). These adult cutoffs were applied consistently in the main age-eligible cohort. Among the 903 participants, 97 were aged 10–17 years and 806 were adults; the younger group comprised 1 OB, 9 OW, 60 NW, and 27 UW participants. Adult BMI cutoffs do not account for age- and sex-specific growth. We therefore performed adult-only sensitivity analyses of ferritin, the cortisol/ACTH ratio, FT4, and recovery, including additional adjusted ferritin and recovery evaluations, but these sensitivity analyses did not validate adult cutoffs in younger participants. Recovery analyses compared NW with OB participants; they did not test a continuous BMI dose–response relationship.

### 2.3. Laboratory Markers

Blood samples were obtained as clinically indicated, primarily in the late morning after at least 5 min of seated rest. Sampling times were not standardized to a fixed morning window, and fasting status and a reliable onset-to-blood interval were unavailable. Glucose values were not assumed to be fasting measurements. Free thyroxine (FT4, ng/dL), adrenocorticotropic hormone (ACTH, pg/mL), cortisol (µg/dL), and ferritin (ng/mL) were measured with the Cobas 8000 system (F. Hoffmann-La Roche AG, Basel, Switzerland) at the Central Laboratory of Okayama University Hospital. Other recorded markers included blood counts, albumin, liver enzymes, renal markers, lipids, glucose, electrolytes, and thyroid-stimulating hormone (TSH). The cortisol/ACTH ratio was calculated as serum cortisol divided by plasma ACTH; it was used descriptively and was not interpreted as a diagnostic test of hypothalamic–pituitary–adrenal function. Ferritin was evaluated in the total biomarker cohort and by recorded sex. FT3, thyroid antibodies, cortisol-binding globulin, complete iron indices, and fully standardized sampling data were not consistently available. Adult (not pediatric) institutional reference intervals applicable during the study period are provided in Table S1, and no validated reference interval was assigned to the derived cortisol/ACTH or FT4/TSH ratios. Laboratory-value availability within the medication-excluded cohort is summarized in Table S1; demographic comparisons are reported in Section 3.1.

### 2.4. Major Symptoms Related to Long COVID

Major presenting symptoms were elicited and documented by the treating physicians during clinical assessment and retrospectively extracted from the structured clinical records. Standardized symptom-severity or functional scores were not available for every record. The analysis categories were fatigue, brain fog, headache, sleep disturbance, decreased concentration, dyspnea, fatigability, dizziness, olfactory disturbance, gustatory disturbance, fever, palpitations, and cough. These symptoms were not mutually exclusive.

### 2.5. Statistical Analysis

Continuous variables are summarized as medians and interquartile ranges, and categorical variables as counts and percentages. We used Mann–Whitney U and chi-square tests to compare baseline characteristics by recorded sex and Kruskal–Wallis and chi-square tests to compare them across BMI categories. Baseline laboratory comparisons used Kruskal–Wallis tests followed by pairwise Mann–Whitney U tests with Bonferroni correction; superscript letters in the tables below identify adjusted differences. Analyses were conducted in Python 3.13.9 using NumPy 2.3.5, pandas 2.3.3, SciPy 1.16.3, statsmodels 0.14.5, lifelines 0.30.3, and openpyxl 3.1.5; Python plots used Matplotlib 3.10.6.

For evaluating focused biomarker distributions, four-group Kruskal–Wallis tests were followed by two-sided Mann–Whitney U comparisons of UW, OW, and OB with NW participants, with Bonferroni correction for three comparisons regarding each marker. Analyses comparing all BMI category pairs used the corresponding six-comparison correction. Log-transformed linear regression models used NW as the reference and adjusted for age and recorded sex; sex-stratified ferritin models adjusted for age. Adult-only rank-based sensitivity analyses assessed ferritin, the cortisol/ACTH ratio, and FT4. The WHO-compatible sensitivity analysis repeated rank-based biomarker comparisons, adjusted log-biomarker model evaluations, and recovery analyses without replacing the main clinic-cohort assessments. The study had an exploratory objective, not a single prospectively specified confirmatory primary endpoint. Regression, symptom, subgroup, and sensitivity analyses were exploratory. Nominal and within-marker Bonferroni-adjusted *p* values are distinguished; pointwise 95% confidence intervals do not provide study-wide multiplicity control. Descriptive biomarker summaries for the main cohort and adjusted estimates for the WHO-compatible cohort are presented in Table S2.

Additional male ferritin analyses used linear regression of natural-log ferritin with BMI category indicators, age per 10 years, past/current smoking, and habitual alcohol use (Table S3A). HC3 heteroskedasticity-consistent standard errors and t-based inference provided pointwise confidence intervals; exponentiated BMI coefficients are ratios of modeled geometric means. Separate sensitivities restricted the sample to adults, added recorded acute severity (mild versus moderate/severe), or added log alanine aminotransferase (ALT) and log triglycerides. For expanded models, base models were also fitted among the same participants with complete data. The three BMI contrasts with NW were Bonferroni-adjusted within each model. No missing values were imputed; sample sizes reflect the data available for each marker and model. Diagnoses were not inferred from biomarkers, and covariates or observations were not selected according to *p* values.

Kaplan–Meier curves estimated cumulative recovery from symptom onset, with recovery as the event, and log-rank tests compared NW and OB within recorded-sex strata. The recovery cohort included 565 participants with complete dates. A pooled Cox proportional-hazards model included obesity, recorded sex, obesity × sex, and age per 10 years; female sex and NW were reference categories, and Efron’s method was used to handle ties. Hazard ratios (HRs) greater than 1 indicate a higher instantaneous recovery rate. A further pooled complete-case model added past/current smoking and habitual alcohol use. Proportional-hazards assumptions were assessed using Schoenfeld residuals. Recovery analyses were exploratory; the additional regression and sensitivity analyses were conducted during revision.

Sensitivity analyses included restriction to adults, an onset-based model with delayed entry at the first visit to the CAC clinic, and models using the first visit as the time origin. The WHO-compatible recovery sensitivity analysis used symptom onset as the time origin. Model-specific sample sizes, exclusions, and proportional-hazards diagnostics are reported in Table S3B.

## 3. Results

### 3.1. Study Cohort and Baseline Characteristics

Of 908 screened clinic attendees with BMI data, 5 were younger than 10 years, leaving 903 participants: UW 130, NW 525, OW 174, and OB 74. The median age was 42.0 years [IQR 27.0–53.0], 473 participants were female, and 97 participants were aged 10–17 years (Table 1). The biomarker cohort comprised 755 participants after the medication-related exclusions described above. Baseline characteristics differed across BMI categories for age, smoking, alcohol use, oxygen therapy, and onset-to-first-visit interval, whereas vaccination status and hospital admission did not (Table 2). Table 3 reports cohort denominators and marker- and BMI-specific available counts. These differences and the single-center referral setting were considered when interpreting exploratory associations.

Past/current smoking was recorded for 891/903 participants and habitual alcohol use for 890/903. Acute COVID-19 severity was classified in 897 participants: 861 mild, 19 moderate I, 10 moderate II, and 7 severe. The number of vaccine doses received before the index SARS-CoV-2 infection was recorded for 886/903 participants. Table S1 reports laboratory-value availability within the 755-person biomarker cohort. For ferritin, 647 participants had a value and 108 did not. Their median ages were 42 versus 37 years, the proportions recorded as male were 48.8% versus 46.3%, and the median BMIs were 22.04 versus 22.21 kg/m^2^. Standardized differences in mean age, male proportion, and mean BMI were 0.055, 0.051, and −0.041, respectively. Across the listed markers and ratios, the largest absolute standardized differences were 0.131 for age, 0.087 for BMI, and 0.066 for male proportion.

### 3.2. Major Long COVID Symptoms Stratified by BMI Category

**Figure 2:**
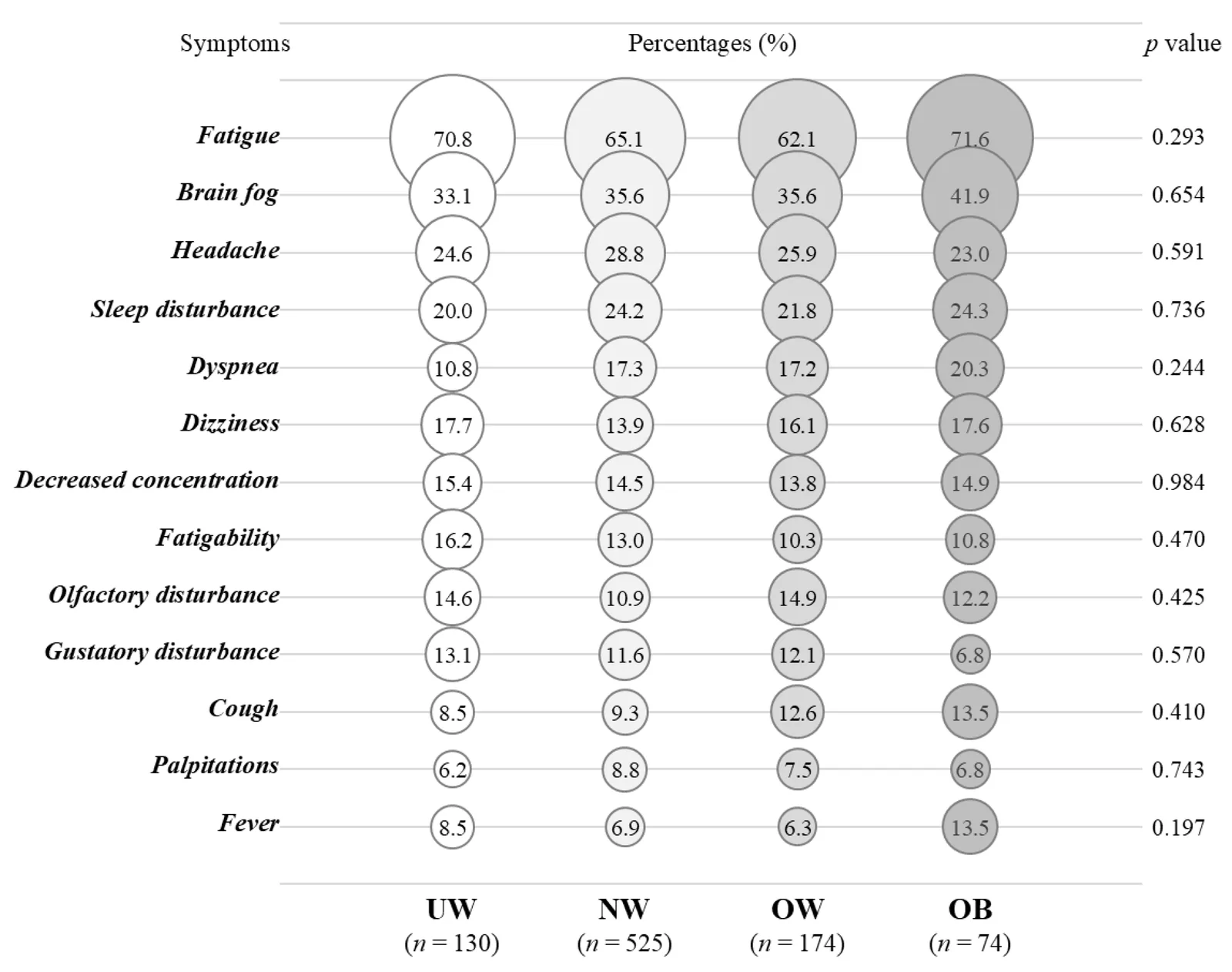
**Dot plots of symptoms presented across BMI categories.** Dot size and labels indicate the percentage of participants with each symptom. Four-group chi-square tests were interpreted descriptively because multiple symptoms were evaluated. The eligible cohort comprised the following numbers of participants: UW: *n* = 130; NW: *n* = 525; OW: *n* = 174; and OB: *n* = 74. Symptoms are ordered by overall prevalence and were not mutually exclusive. BMI category definitions are provided in the Figure 1 legend.

Presenting symptoms were broadly similar across BMI categories (Figure 2). Fatigue and brain fog were common in every group. Because symptom categories were not mutually exclusive, derived from clinical records, and tested across multiple categories, these comparisons are descriptive and do not exclude residual differences in symptom severity or ascertainment.

### 3.3. Ferritin Profiles Across BMI Categories

**Figure 3:**
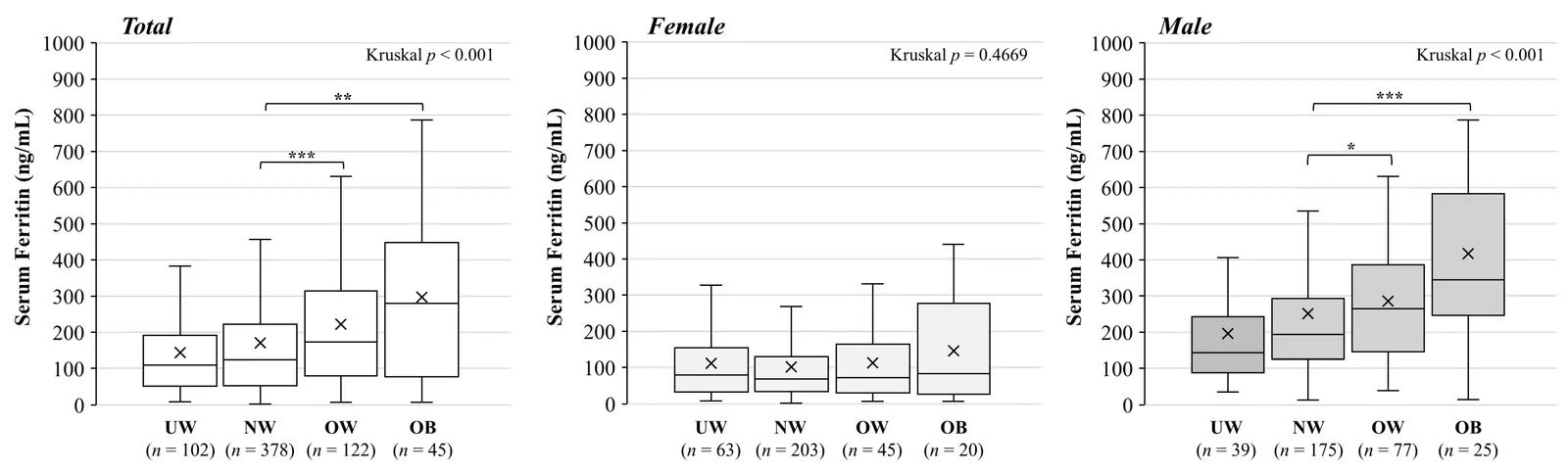
**Ferritin profiles across BMI categories.** Boxplots show median, interquartile range, whiskers at 1.5 × IQR, and mean (×) in the total cohort and by recorded sex. Marker-specific sample sizes are total UW 102, NW 378, OW 122, and OB 45; female UW 63, NW 203, OW 45, and OB 20; and male UW 39, NW 175, OW 77, and OB 25. Kruskal–Wallis tests assessed four-group differences. Mann–Whitney U comparisons with NW were Bonferroni-adjusted within each panel (* *p* < 0.05, ** *p* < 0.01, *** *p* < 0.001). Boxplots retain the original exclusive-quartile convention; tabulated IQRs in Table S2A use linear interpolation. BMI category definitions are provided in the Figure 1 legend.

Ferritin was available for 647 participants: UW 102, NW 378, OW 122, and OB 45 (Figure 3). Median [IQR] ferritin levels were 109.0 [52.7–189.5], 124.0 [53.1–220.5], 173.5 [80.9–312.2], and 280.0 [82.1–440.0] ng/mL, respectively (global *p* < 0.001). Bonferroni-adjusted comparisons with NW were significant for OW (*p* = 0.000614) and OB (*p* = 0.00188), but not UW. Descriptive results by BMI category and recorded sex are provided in Table S2A. The association was predominantly observed among men (*n* = 316; global *p* < 0.001; OW vs. NW *p* = 0.0436; OB vs. NW *p* = 0.000602), whereas no clear pattern was observed among women (*n* = 331; global *p* = 0.467). In the limited adult-only sensitivity analysis of the assay dataset, the male OB versus NW contrast remained significant (*p* = 0.000636), whereas OW versus NW did not (*p* = 0.0938). The obesity-associated ferritin signal among men remained evident in the adult-only sensitivity analysis, although the pattern was not uniformly significant across all BMI categories.

### 3.4. Endocrine Biomarker Profiles Across BMI Categories

The cortisol/ACTH ratio was available for 644 participants (UW/NW/OW/OB *n* = 100/377/122/45); corresponding medians were 0.420/0.336/0.296/0.298 (global *p* < 0.001). Among women (*n* = 331), the medians were 0.498/0.422/0.385/0.329 (global *p* = 0.0043); although the ratio was lower in the high-BMI groups, no significant differences were observed in any of the comparisons against the NW group after Bonferroni correction. The four-group comparison among male participants was not significant (*n* = 313; *p* = 0.0654).

**Figure 4:**
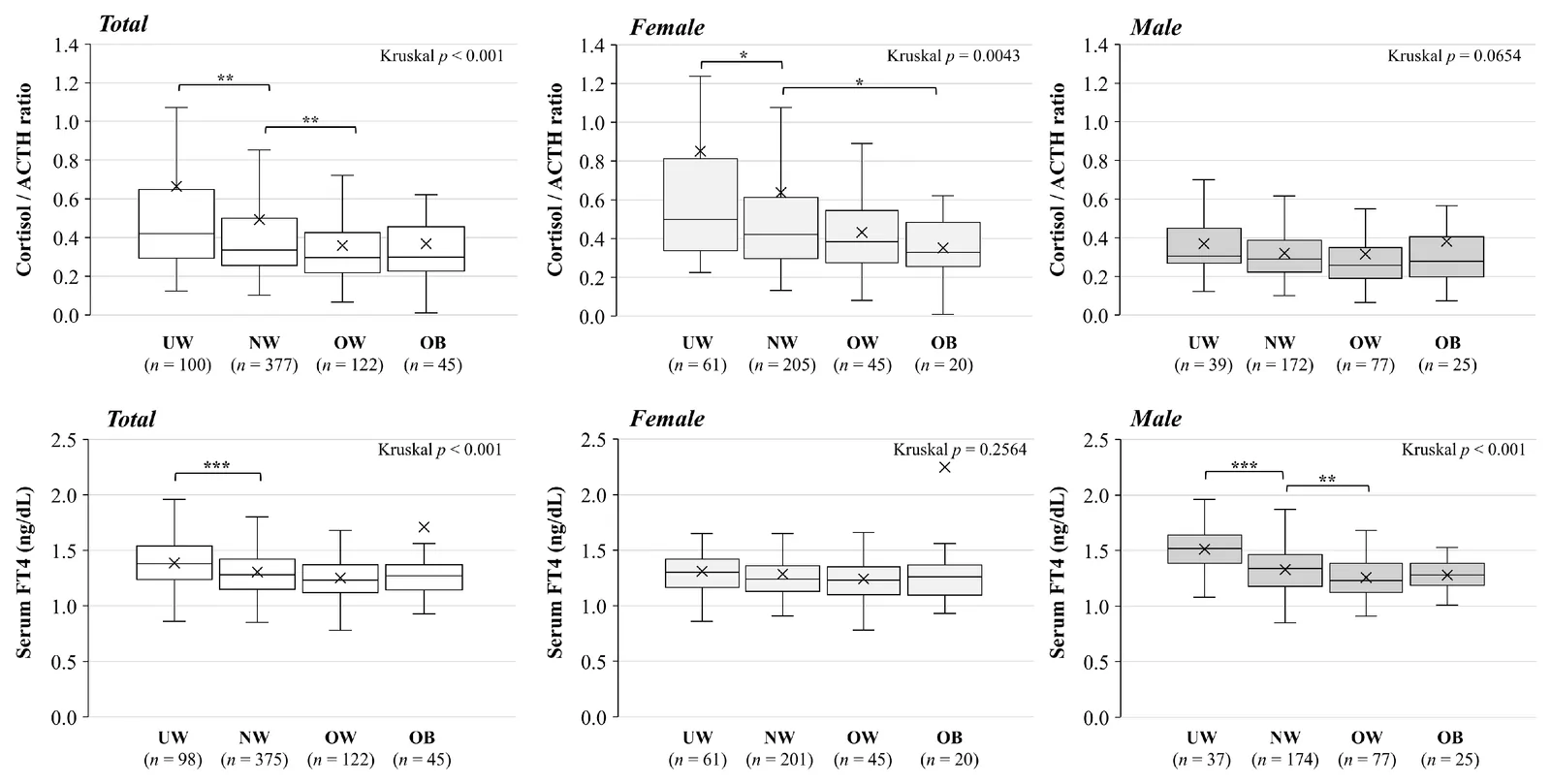
**BMI-associated endocrine biomarker patterns.** Boxplots show cortisol/ACTH ratio and FT4 in the total cohort and by recorded sex. Boxes indicate the IQR with a median line; × denotes the mean. Boxplots retain the original exclusive-quartile convention; tabulated IQRs in Table S2A use linear interpolation. Sample sizes were cortisol/ACTH ratio UW 100, NW 377, OW 122, and OB 45 (total *n* = 644) and FT4 UW 98, NW 375, OW 122, and OB 45 (total *n* = 640). Kruskal–Wallis and within-marker Bonferroni-adjusted Mann–Whitney U comparisons with NW were used (* *p* < 0.05, ** *p* < 0.01, *** *p* < 0.001). These single measurements were obtained as clinically indicated, without fully standardized collection times. They should not be interpreted as diagnostic endocrine-function tests. BMI category definitions are provided in the Figure 1 legend.

FT4 was available for 640 participants (*n* = 98/375/122/45); medians were 1.38/1.28/1.23/1.27 ng/dL (global *p* < 0.001). In men (*n* = 313), FT4 medians were 1.52/1.34/1.23/1.28 ng/dL (global *p* < 0.001), with higher FT4 in UW than NW (Bonferroni *p* < 0.001) and lower FT4 in OW than NW (*p* = 0.0173), but no OB–NW difference. The four-group comparison among female participants was not significant (*n* = 327; *p* = 0.2564). These descriptive patterns do not establish a universal BMI dose–response or a sex interaction (Figure 4; Table S2A).

### 3.5. Adjusted Biomarker Models

**Figure 5:**
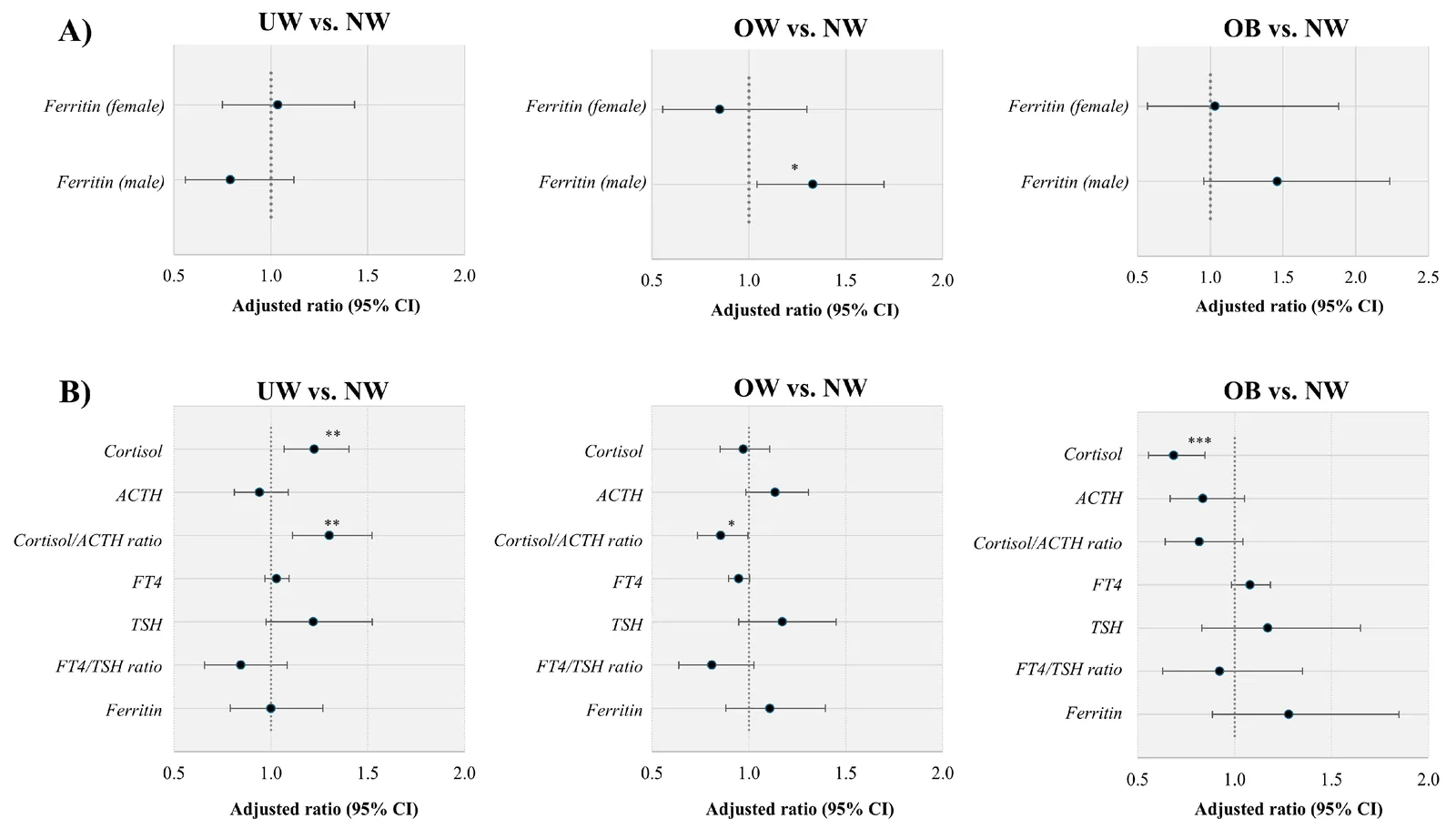
**Adjusted biomarker ratios in the WHO-compatible sensitivity cohort.** Of 572 participants, 484 remained eligible after corticosteroid and thyroid hormone exclusions. Panel (**A**) shows age-adjusted ferritin ratios in women (*n* = 214) and men (*n* = 201). Panel (**B)** shows age- and recorded-sex-adjusted models of log-transformed biomarkers: cortisol, ACTH, and the cortisol/ACTH ratio (each *n* = 413), FT4 (*n* = 410), TSH and the FT4/TSH ratio (each *n* = 409), and ferritin (*n* = 415). NW is the reference. Points and error bars show geometric-mean ratios and pointwise 95% confidence intervals. Asterisks indicate nominal exploratory regression *p* values (* *p* < 0.05, ** *p* < 0.01, *** *p* < 0.001), not the Bonferroni-adjusted rank-based comparisons reported in Section 3.7. All numerical results are provided in Table S2B. These sensitivity models do not replace the main clinic-cohort analyses. BMI category definitions are provided in the Figure 1 legend.

Figure 5 presents adjusted biomarker models in the WHO-compatible sensitivity cohort of 572 participants. After the same corticosteroid and thyroid hormone exclusions as in the main biomarker analysis, 484 participants were eligible, with available-case sample sizes varying by marker. In the age-adjusted ferritin models, the male OW-versus-NW ratio was 1.330 (95% CI: 1.042–1.697; *n* = 201; nominal *p* = 0.02229), and the male OB-versus-NW ratio was 1.461 (95% CI: 0.955–2.234; nominal *p* = 0.08019). None of the female ferritin contrasts was statistically significant (*n* = 214). Panel B reports the results of the age- and recorded-sex-adjusted models, while Table S2B provides all estimates, pointwise confidence intervals, nominal *p* values, and model-specific sample sizes. These WHO-compatible sensitivity models are distinct from the main-cohort HC3 analyses in Table S3A.

In the main clinic cohort, among 313 men with ferritin and lifestyle data, the age-, smoking-, and alcohol-adjusted OB-versus-NW geometric-mean ratio was 1.680 (95% CI: 1.125–2.510; nominal *p* = 0.01148; Bonferroni-adjusted *p* = 0.03443). Adding acute severity gave a ratio of 1.682 (95% CI: 1.126–2.513; adjusted *p* = 0.03365), although only 14 men in this model had moderate/severe acute illness. In 269 adult men, the corresponding base-model ratio was 1.686 (95% CI: 1.147–2.477; adjusted *p* = 0.02406).

In 298 men with ALT and triglyceride measurements, the OB-versus-NW ratio was 1.886 (95% CI: 1.373–2.590) in the same-sample base model and 1.211 (95% CI: 0.843–1.741) (nominal *p* = 0.2997; adjusted *p* = 0.899) after adding log ALT and log triglycerides. The conditional obesity estimate was reduced after including these hepatic/metabolic correlates; however, this analysis does not establish independence from these correlates or causal mediation. All BMI contrasts and models are reported in Table S3A.

### 3.6. Association Between BMI and Recovery

**Figure 6:**
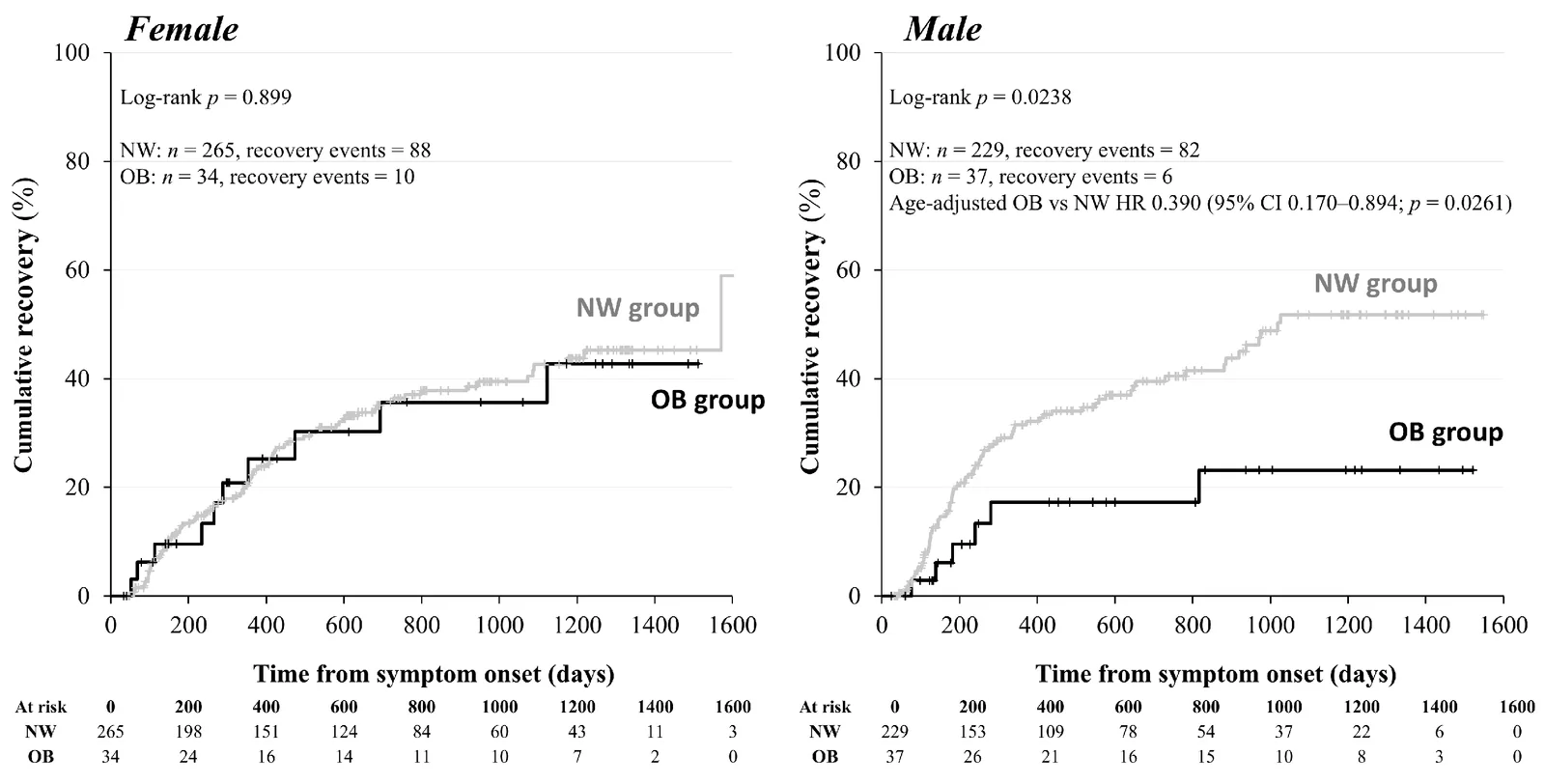
**Cumulative recovery from symptom onset by recorded sex.** Kaplan–Meier curves compare NW and OB participants in the 565-person recovery cohort; numbers at risk are shown at 200-day intervals. Recovery was defined as the point at which the attending physician determined that care at the CAC clinic should be concluded due to sufficient symptom improvement; complete disappearance of symptoms was not a requirement. Participants who had not recovered and remained under follow-up were censored on 31 March 2026; those referred elsewhere or lost to follow-up were censored at the last known contact. Men: NW *n* = 229/events = 82; OB *n* = 37/events = 6; log-rank *p* = 0.0238. Women: NW *n* = 265/events = 88; OB *n* = 34/events = 10; log-rank *p* = 0.8994. The age-adjusted pooled Cox model included 565 participants/186 events; the HR for obesity versus normal weight among male participants was 0.390 (95% CI: 0.170–0.894; *p* = 0.0261). The obesity × sex interaction was not significant (*p* = 0.0866); sex-specific effect modification was not established. First-visit-based and delayed-entry results are reported in Table S3B. BMI category definitions are provided in the Figure 1 legend.

In the main analysis, using symptom onset as time zero, men with obesity had a longer time to clinically defined recovery than normal-weight men (log-rank *p* = 0.0238). Kaplan–Meier analysis included 565 participants (494 NW and 71 OB) and 186 recovery events. NW included 229 participants with 82 events among men and 265 participants with 88 events among women, while OB included 37 participants with 6 events among men and 34 participants with 10 events among women (log-rank *p* = 0.8994) (Figure 6). The age-adjusted pooled Cox model included 565 participants and 186 events. The HR for OB versus NW among male participants was 0.390 (95% CI: 0.170–0.894; nominal *p* = 0.0261), with an interaction *p* = 0.0866. The association among male participants remains exploratory; different subgroup *p* values do not establish effect modification by sex.

After additional adjustment for smoking and habitual alcohol use, the onset-based pooled model included 555 participants and 185 events. The HR for obesity versus normal weight among male participants was 0.378 (95% CI: 0.165–0.868; nominal *p* = 0.02176), with an interaction *p* = 0.06122. The age-adjusted model fitted to the same 555 participants gave an HR of 0.388 (95% CI: 0.169–0.889). Only six recovery events occurred among men with obesity in these models (Table S3B).

The adult-only recovery sensitivity analysis used symptom onset as the time origin and included 505 participants and 159 events. The male and female log-rank *p* values were 0.0423 and 0.9192, respectively. In the pooled age-adjusted model restricted to adults, the HR for obesity versus normal weight among men was 0.426 (95% CI: 0.185–0.982; nominal *p* = 0.04516), with an interaction *p* = 0.1048. Adult men with obesity still contributed only six recovery events. Table S3B summarizes the pooled models.

In supplementary analyses using the first visit to the CAC clinic as time zero, the age-adjusted pooled model included 564 participants and 186 events and gave an HR of 0.338 for OB versus NW among male participants (95% CI: 0.147–0.774; *p* = 0.01033; interaction *p* = 0.05980). Adding smoking and alcohol resulted in an HR of 0.328 (95% CI: 0.143–0.752; *p* = 0.008468; interaction *p* = 0.03964) with 554 participants and 185 events. With symptom onset as time zero and delayed entry at first visit, the age-adjusted model produced an HR of 0.304 (95% CI: 0.133–0.697; *p* = 0.004915; interaction *p* = 0.06304); the corresponding lifestyle-adjusted model gave an HR of 0.302 (95% CI: 0.132–0.693; *p* = 0.004733; interaction *p* = 0.03962). The direction of the association persisted across these specifications, all based on the same six recovery events among men with obesity. These exploratory analyses are not independent replications (Table S3B).

### 3.7. WHO Clinical-Compatibility Sensitivity Analysis

The WHO-compatible sensitivity cohort was a nested subset of the main clinic cohort, comprising 572 of 903 patients (63.3%). The cohort comprised 311 women and 261 men, with a median age of 41 years [IQR: 27–53] and UW/NW/OW/OB counts of 88/335/113/36. The longitudinal and clinical eligibility criteria are described in Section 2.1.

After the unchanged medication exclusions, the WHO-compatible biomarker cohort comprised 484 participants, with ferritin information available for 415. Among men, the ferritin medians were 110.5, 187.0, 266.0, and 306.0 ng/mL for UW, NW, OW, and OB, respectively (global *p* = 0.000263); the corresponding sample sizes were 22, 111, 55, and 13. The OW-versus-NW comparison remained significant after within-marker Bonferroni correction (*p* = 0.01273); the OB-versus-NW comparison did not reach this threshold (*p* = 0.07626). Thus, the overall male BMI group difference remained detectable, without establishing an obesity-specific contrast. Adjusted models for this restricted, WHO-compatible cohort are shown separately in Figure 5 and Table S2B; their nominal regression *p* values are distinct from the rank-based comparisons above.

The WHO-compatible recovery analysis used symptom onset as time zero and included 359 participants and 107 recovery events. The log-rank *p* values were 0.177 overall, 0.146 in men (NW: 144 participants/43 events; OB: 18/2), and 0.677 in women (NW: 181/58; OB: 16/4). The main and WHO-compatible recovery analyses retained their respective eligibility criteria, including available symptom-onset and follow-up dates and restriction to the NW/OB comparison.

## 4. Discussion

### 4.1. Main Findings and Clinical Interpretation

In this retrospective specialist-clinic cohort study, we recorded BMI-related endocrine and metabolic biomarker patterns, alongside an association between obesity and a longer time to clinically defined recovery among men. This association was observed in the main Kaplan–Meier analysis, the age-adjusted pooled Cox model, and additional analyses with lifestyle adjustment, restriction to adults, delayed entry, and first-visit time as the origin. This finding is clinically relevant as a hypothesis for further study, but six recovery events among men with obesity limit precision. The main onset-based age-adjusted sex-by-obesity interaction was not significant, and additional time-origin interaction results varied by adjustment. Thus, a causal effect or stable sex-specific difference is not established.

Ferritin, an inflammatory marker hypothesized to be linked to Long COVID, is an iron-storage protein and a non-specific acute-phase reactant [44]. Its relationship with Long COVID therefore needs to be interpreted in the context of adiposity and metabolic inflammation [14]. Our earlier study found higher ferritin in women with ME/CFS than in women without in a largely pre-Omicron cohort [44]. In the phase-stratified analysis by Morita et al., this female ME/CFS-related contrast was detected in a preceding phase but not in the Delta- or Omicron-dominant phase [45]. These ME/CFS studies provide background on ferritin; ME/CFS was not an outcome or stratification variable in the present study. Our analysis instead examines BMI-associated ferritin patterns within a symptomatic clinic cohort, predominantly among men. Differences in period, case mix, and outcome definition may contribute to the differing observations; the earlier reports do not establish a mechanism or validate the present BMI association. The male obesity contrast persisted after adjustment for age, smoking, and alcohol and in the adult-only analysis, while additional adjustment for ALT and triglycerides reduced the conditional obesity estimate in the same patients. These correlates may also lie on obesity-related pathways, so this is not a causal mediation analysis. The results characterize BMI-associated clinical biochemistry, not a Long COVID-specific ferritin mechanism or an explanation for delayed recovery.

Previous studies have associated obesity with the risk of developing Long COVID [4,6,7,8,9]. Our study addresses a different question: how BMI relates to clinical biomarkers and subsequent recovery among patients already attending a Long COVID clinic. Acute COVID-19 studies also suggest that host associations can vary by sex and pandemic phase [47,48], but these findings from acute care in hospitals are not direct evidence of Long COVID effect modification. Our sex-stratified observations therefore require replication in adequately powered longitudinal cohorts.

The pooled age-adjusted recovery model gave an HR of 0.390 for obesity versus normal weight among male participants; adding smoking and habitual alcohol use gave an HR of 0.378. Acute severity was available in the source records, yet only five events occurred among 20 participants with moderate/severe acute illness in the recovery cohort. Recovery reflected physician-determined conclusion of outpatient follow-up after sufficient symptom improvement rather than complete symptom resolution. This approach is consistent with published methodology from the same CAC clinic setting in a previous study [46], but that report is procedural support rather than independent external validation. Endpoints based on the conclusion of outpatient follow-up are influenced by clinical practice, patient preferences, access to healthcare, and the potential for “informative censoring”; consequently, comparisons with other studies using symptom resolution or functional recovery as endpoints are limited. Nevertheless, a key finding of this exploratory study is that men with obesity had a significantly longer time to clinically defined recovery than normal-weight men in the main clinic cohort.

The endocrinological findings supporting these observations complement the metabolic data presented here. While differences in the cortisol/ACTH ratio across BMI categories were observed in women, differences in FT4 levels were more pronounced in men. Median cortisol/ACTH ratios in female participants declined across BMI categories, but individual NW-referenced comparisons were not significant after multiplicity correction. This distinction supports a descriptive group-level pattern rather than an obesity-specific endocrine abnormality. Our earlier research linked basal endocrine measurements to symptom profiles [25], providing context for studying these measures without establishing diagnostic validity. Here, a single basal ratio reflects measured cortisol relative to ACTH at one clinical assessment; it is not a dynamic measure of adrenal responsiveness. A separate exploratory stimulation-test study of 16 highly selected patients with refractory Long COVID reported largely preserved peak anterior pituitary responses with delayed response kinetics [31], but that study had no general control group and does not validate the present BMI patterns. These observations justify further standardized endocrine investigation, but not a diagnosis of hypothalamic–pituitary–adrenal dysfunction or chronic glucocorticoid excess.

In addition, the male FT4 pattern was characterized chiefly by higher levels in underweight men and lower levels in overweight men relative to normal-weight men, rather than a uniform decrease across increasing BMI categories. This may reflect variation in nutritional and metabolic context, but the present cross-sectional data cannot determine its direction or cause. Thyroid measurements during acute COVID-19 have differed from pre-infection measurements and returned toward baseline at follow-up [30], but this acute-care evidence is contextual, not proof of persistent thyroid disease in this cohort. We therefore retain the term BMI-associated endocrine biomarker pattern. Clinical assessment and repeat measurements remain appropriate when indicated.

### 4.2. Case Definition and Limitations

The one-month clinic criterion used in our study included patients evaluated earlier than the WHO post-COVID-19 condition time frame [1]. The 572-patient WHO-compatible cohort provides a case-definition sensitivity analysis based on longitudinal symptom trajectories and clinical assessment, without replacing the main clinic cohort. Within this cohort, ferritin differed across BMI groups in men, but interpreting the recovery analysis is limited by the small subgroup event counts. NICE also uses Long COVID as an umbrella term encompassing ongoing symptomatic COVID-19 from 4 to 12 weeks and post-COVID-19 syndrome beyond 12 weeks [49]. This provides context for studying an earlier symptomatic clinic population but does not make the one-month operational criterion equivalent to the WHO PCC definition.

BMI may provide context for interpreting routine laboratory findings in this clinic cohort, but neither BMI nor these biomarkers were validated here as standalone diagnostic or prognostic tools for Long COVID. Clinical assessment and investigations should be guided by the patient’s symptoms and presentation, including possible alternative diagnoses, irrespective of BMI [49]. In addition, symptoms were documented by physicians in this study, but a uniform severity or functional scale was not available for all patients.

In the present study, several additional limitations should be acknowledged. This retrospective single-center specialist-clinic referral cohort may overrepresent persistent, complex, or care-seeking cases and may not generalize to community populations. Sampling times were not standardized, and a reliable onset-to-blood interval was unavailable. Information on fasting status, reinfection, physical activity, body composition, menstrual or menopausal status, FT3, thyroid antibodies, cortisol-binding globulin, and complete iron indices was incomplete or unavailable. Prescription records covered different treatment periods, and blanks could not be treated as absence of exposure. Adult-only analyses did not validate adult BMI thresholds in younger participants. Sparse events, potentially influential ferritin observations, clinically indicated testing, and medication exclusions limited precision and may have introduced bias. The main onset-based analysis did not account for delayed entry. Supplementary delayed-entry and first-visit-based analyses addressed the timing of clinic entry but did not remove clinic-selection bias, establish independent truncation, or rule out informative censoring. Multiple analyses and nominal *p* values increased the chance of false-positive findings. Finally, the clinically defined recovery endpoint was not a validated symptom or functional scale, and residual confounding by comorbidities and medication use remained possible.

## 5. Conclusions

In conclusion, men with obesity in the main symptomatic clinic cohort had a longer time to clinically defined recovery than normal-weight men, and BMI categories were associated with inflammatory and/or endocrine biomarker patterns, including cortisol/ACTH ratios in women and ferritin and FT4 in men. The recovery association was also observed in additional analyses with limited lifestyle adjustment, restriction to adults, delayed entry, and first-visit time as the origin. The main interaction was not significant and additional interaction results were specification-dependent; in addition, the WHO-compatible recovery analysis was limited by sparse events, and hepatic/metabolic adjustment reduced the conditional ferritin estimate. These exploratory findings support further investigating obesity and recovery, without establishing causality, a universal BMI dose–response relationship, or a definitive male-specific effect. Larger prospective studies with adequate numbers of recovery events, standardized measurements, and detailed confounder assessment are needed.

## Figures and Tables

**Table 1:** Clinical characteristics of the analytic cohort stratified by sex.

Characteristics	Overall (*n* = 903)	Female (*n* = 473)	Male (*n* = 430)	*p-*Value
Age, years (median [IQR])	42.0 [27.0–53.0]	43.0 [28.0–53.0]	41.0 [25.2–53.0]	0.2548
BMI, kg/m^2^ (median [IQR])	22.2 [19.8–25.5]	21.3 [19.2–24.5]	23.4 [20.8–26.3]	<0.001 ***
BMI category: Underweight (<18.5 kg/m^2^)	130 (14.4%)	84 (17.8%)	46 (10.7%)	<0.001 ***
BMI category: Normal weight (18.5–24.9 kg/m^2^)	525 (58.1%)	282 (59.6%)	243 (56.5%)	<0.001 ***
BMI category: Overweight (25.0–29.9 kg/m^2^)	174 (19.3%)	72 (15.2%)	102 (23.7%)	<0.001 ***
BMI category: Obesity (≥30.0 kg/m^2^)	74 (8.2%)	35 (7.4%)	39 (9.1%)	<0.001 ***

Values are shown as median [IQR] or *n* (%). Percentages were calculated using available data for each sex. *p* values were calculated using the Mann–Whitney U test for continuous variables and the chi-square test for categorical variables. For BMI category, a single overall *p* value was calculated for the distribution across categories and repeated across the corresponding rows. Asterisks indicate nominal statistical significance (*** *p* < 0.001). Gray shading denotes table headings only.

**Table 2:** Clinical characteristics of the analytic cohort stratified by BMI category.

Patient Groups	UW (*n* = 130)	NW (*n* = 525)	OW (*n* = 174)	OB (*n* = 74)	*p-*Value
Age, years (median [IQR])	33.0 [19.2–51.0]	40.0 [25.0–53.0]	46.0 [36.0–56.0]	43.5 [34.2–49.8]	<0.001 ***
**Lifestyle**					
Smoking habit, *n* (%)	17 (13.1%)	122 (23.6%)	68 (39.5%)	28 (38.9%)	<0.001 ***
Alcohol drinking, *n* (%)	18 (13.8%)	125 (24.2%)	53 (30.8%)	22 (30.6%)	0.0044 **
**COVID-19 vaccination status**					
<1 doses, *n* (%)	31 (24.0%)	104 (20.2%)	40 (23.5%)	23 (31.5%)	0.1577
≥1 doses, *n* (%)	98 (76.0%)	410 (79.8%)	130 (76.5%)	50 (68.5%)	0.1577
**Clinical severity in the acute phase of COVID-19**					
Hospital admission, *n* (%)	12 (9.2%)	30 (5.7%)	13 (7.5%)	7 (9.5%)	0.3836
Oxygen therapy, *n* (%)	0 (0.0%)	8 (1.5%)	7 (4.0%)	3 (4.1%)	0.0362 *
Mild condition, *n* (%)	126 (96.9%)	506 (96.9%)	162 (93.1%)	69 (94.5%)	0.1278
Moderate to severe condition, *n* (%)	4 (3.1%)	16 (3.1%)	12 (6.9%)	4 (5.5%)	0.1278
**Duration from the onset to the first visit**					
Days: median [IQR]	112.0 [73.0–219.0]	107.0 [66.0–230.0]	114.0 [70.0–222.0]	86.0 [51.5–149.5]	0.0363 *

Values are shown as median [IQR] or *n* (%). Vaccination status refers to doses received before the index SARS-CoV-2 infection. Percentages were calculated using available data within each BMI category. *p* values were calculated using the Kruskal–Wallis test for continuous variables and the chi-square test for categorical variables. Baseline comparisons were interpreted descriptively. Asterisks indicate nominal statistical significance (* *p* < 0.05, ** *p* < 0.01, *** *p* < 0.001). BMI category definitions are provided in the Figure 1 legend. Gray shading denotes table headings only.

**Table 3:** Baseline laboratory findings stratified by BMI category.

Biomarker (Median [IQR])	UW (*n* = 113)	NW (*n* = 450)	OW (*n* = 137)	OB (*n* = 55)	*p*-Value
Hemoglobin, g/dL	13.9 [13.4–15.1] ^a^ *n* = 102	14.2 [13.3–15.3] ^a^ *n* = 383	15.1 [14.3–15.9] ^b^ *n* = 124	14.7 [13.8–15.8] ^ab^ *n* = 45	<0.001 ***
Albumin, g/dL	4.6 [4.3–4.8] ^a^ *n* = 102	4.5 [4.3–4.7] ^ab^ *n* = 378	4.4 [4.2–4.6] ^b^ *n* = 122	4.4 [4.1–4.5] ^b^ *n* = 45	<0.001 ***
AST, U/L	18.0 [15.0–22.0] ^a^ *n* = 101	18.0 [15.0–22.0] ^a^ *n* = 381	22.0 [17.0–28.0] ^b^ *n* = 123	33.0 [22.8–44.0] ^c^ *n* = 44	<0.001 ***
ALT, U/L	14.0 [11.0–20.0] ^a^ *n* = 101	16.0 [11.0–23.0] ^a^ *n* = 381	26.0 [18.0–44.5] ^b^ *n* = 123	49.5 [27.5–78.2] ^c^ *n* = 44	<0.001 ***
γ-GTP, U/L	18.0 [13.0–24.2] ^a^ *n* = 100	19.0 [14.0–30.0] ^a^ *n* = 380	32.0 [22.0–55.5] ^b^ *n* = 123	56.5 [34.8–78.8] ^c^ *n* = 44	<0.001 ***
BUN, mg/dL	11.6 [9.5–13.2] ^a^ *n* = 101	12.3 [10.3–14.6] ^a^ *n* = 380	12.5 [9.9–15.1] ^a^ *n* = 123	11.6 [9.6–15.1] ^a^ *n* = 44	0.0640
Creatinine, mg/dL	0.67 [0.58–0.77] ^a^ *n* = 101	0.71 [0.59–0.82] ^a^ *n* = 380	0.78 [0.66–0.89] ^b^ *n* = 123	0.72 [0.63–0.86] ^ab^ *n* = 44	<0.001 ***
HDL-C, mg/dL	69.0 [60.0–86.0] ^a^ *n* = 96	65.0 [54.0–79.0] ^b^ *n* = 364	55.0 [47.2–65.0] ^c^ *n* = 118	47.0 [41.2–59.5] ^c^ *n* = 42	<0.001 ***
LDL-C, mg/dL	105.0 [93.0–123.0] ^a^ *n* = 97	111.0 [94.0–134.0] ^a^ *n* = 365	127.0 [109.2–156.0] ^b^ *n* = 118	135.0 [116.5–152.5] ^b^ *n* = 43	<0.001 ***
Triglycerides, mg/dL	70.0 [53.8–99.0] ^a^ *n* = 96	94.0 [65.0–144.0] ^b^ *n* = 363	138.5 [94.2–241.2] ^c^ *n* = 118	204.0 [148.5–309.5] ^c^ *n* = 43	<0.001 ***
Blood glucose, mg/dL	97.0 [91.0–105.0] ^a^ *n* = 101	98.0 [92.0–107.0] ^a^ *n* = 376	104.0 [96.0–114.8] ^b^ *n* = 120	102.5 [96.0–137.0] ^b^ *n* = 44	<0.001 ***

The available sample size for each marker is shown in each cell. Values are shown as median [IQR]. The biomarker cohort included patients aged 10 years or older with available BMI data after excluding patients using corticosteroids or thyroid hormone. Laboratory values were analyzed using available data for each marker. *p* values were calculated using the Kruskal–Wallis test. Post hoc pairwise comparisons among BMI categories were performed using Mann–Whitney U tests with Bonferroni correction. Superscripts a, b, and c denote pairwise-comparison groups. Within each row, values not sharing the same superscript letter are significantly different at *p* < 0.05 after Bonferroni correction. Values sharing at least one superscript letter are not significantly different. Baseline laboratory comparisons were interpreted descriptively. Asterisks indicate nominal statistical significance for the overall Kruskal–Wallis test (*** *p* < 0.001). BMI category definitions are provided in the Figure 1 legend. Gray shading denotes table headings only.

## Data Availability

Detailed data are available upon request from the corresponding authors.

## Supplementary Materials

The following supporting information can be downloaded at: https://www.mdpi.com/article/10.3390/jcm15218492/s1, Table S1: Adult laboratory reference intervals and value availability; Table S2: Descriptive biomarkers and WHO-compatible model results; Table S3: Additional exploratory ferritin and recovery models.

## Abbreviations

ACTH, adrenocorticotropic hormone; BMI, body mass index; CAC, COVID-19 aftercare; CI, confidence interval; FT4, free thyroxine; HR, hazard ratio; OB, obesity; OW, overweight; NW, normal weight; TSH, thyroid-stimulating hormone; UW, underweight.
