# Supplementary material for: Endocrine and Metabolic Characteristics and Prognosis of Long COVID Following SARS-CoV-2 Infection During the Omicron Period
Source: J Clin Med. 2026 Nov 1;15(21):8492. doi: 10.3390/jcm15218492 (PMC13764084; doi:10.3390/jcm15218492)
Supplement: Supplementary file 1 [file jcm-15-08492-s001.zip › jcm-4476517-supplementary.pdf]

Journal of
Clinical Medicine

Supplementary tables:

Table S1. Adult laboratory reference intervals and value availability.

Variable	Unit	Men	Women	Available/missing *n*
Ferritin	ng/mL	39.9–465.0	6.2–138.0	647/108
Cortisol	µg/dL	7.1–19.6	7.1–19.6	644/111
Adrenocorticotropic hormone (ACTH)	pg/mL	7.2–63.3	7.2–63.3	644/111
Free thyroxine (FT4)	ng/dL	0.97–1.69	0.97–1.69	640/115
Thyroid-stimulating hormone (TSH)	mIU/L	0.61–4.23	0.61–4.23	639/116
Hemoglobin	g/dL	13.7–16.8	11.6–14.8	654/101
Albumin	g/dL	4.1–5.1	4.1–5.1	647/108
Aspartate aminotransferase (AST)	U/L	13–30	13–30	649/106
Alanine aminotransferase (ALT)	U/L	10–42	7–23	649/106
γ-Glutamyl transpeptidase	U/L	13–64	9–32	647/108
Creatinine	mg/dL	0.65–1.07	0.46–0.79	648/107
Blood urea nitrogen	mg/dL	8.0–20.0	8.0–20.0	648/107
HDL cholesterol	mg/dL	38–90	48–103	620/135
LDL cholesterol	mg/dL	65–163	65–163	623/132
Triglycerides	mg/dL	40–234	30–117	620/135
Glucose	mg/dL	73–109	73–109	641/114
Cortisol/ACTH ratio	Derived ratio	Not established	Not established	644/111
FT4/TSH ratio	Derived ratio	Not established	Not established	639/116

Reference intervals apply to adults during the study period, not to children, and do not imply fasting sampling. No validated reference intervals were assigned to the derived ratios. Availability refers to recorded values after corticosteroid/thyroid hormone exclusions; each row totals 755. Derived ratios were calculated only when both component measurements were available. Missing values do not establish that a test was never performed. HDL, high-density lipoprotein; LDL, low-density lipoprotein.

Table S2. Descriptive biomarkers and WHO-compatible model results.

A. Main clinic cohort: median [IQR] and available n by BMI and recorded sex.

Marker/group	UW	NW	OW	OB	Global *p*
Ferritin (ng/mL)/Total	109.0 [52.7–189.5]<br>*n* = 102	124.0 [53.1–220.5]<br>*n* = 378	173.5 [80.9–312.2]<br>*n* = 122	280.0 [82.1–440.0]<br>*n* = 45	6.707e-06
Ferritin (ng/mL)/Female	79.4 [33.7–153.0]<br>*n* = 63	68.0 [33.4–129.0]<br>*n* = 203	71.4 [32.5–161.0]<br>*n* = 45	83.0 [37.8–271.8]<br>*n* = 20	0.4669
Ferritin (ng/mL)/Male	143.0 [91.0–239.5]<br>*n* = 39	194.0 [125.0–292.0]<br>*n* = 175	265.0 [146.0–386.0]<br>*n* = 77	345.0 [260.0–530.0]<br>*n* = 25	1.611e-05
Cortisol/ACTH/Total	0.420 [0.294–0.648]<br>*n* = 100	0.336 [0.256–0.500]<br>*n* = 377	0.296 [0.221–0.420]<br>*n* = 122	0.298 [0.242–0.439]<br>*n* = 45	5.761e-06
Cortisol/ACTH/Female	0.498 [0.347–0.769]<br>*n* = 61	0.422 [0.298–0.610]<br>*n* = 205	0.385 [0.287–0.543]<br>*n* = 45	0.329 [0.261–0.454]<br>*n* = 20	0.004327
Cortisol/ACTH/Male	0.306 [0.269–0.438]<br>*n* = 39	0.291 [0.224–0.382]<br>*n* = 172	0.258 [0.190–0.347]<br>*n* = 77	0.278 [0.210–0.336]<br>*n* = 25	0.06544
FT4 (ng/dL)/Total	1.38 [1.24–1.54]<br>*n* = 98	1.28 [1.15–1.42]<br>*n* = 375	1.23 [1.12–1.37]<br>*n* = 122	1.27 [1.16–1.37]<br>*n* = 45	3.661e-05
FT4 (ng/dL)/Female	1.30 [1.17–1.42]<br>*n* = 61	1.24 [1.13–1.36]<br>*n* = 201	1.23 [1.11–1.35]<br>*n* = 45	1.26 [1.10–1.36]<br>*n* = 20	0.2564
FT4 (ng/dL)/Male	1.52 [1.39–1.64]<br>*n* = 37	1.34 [1.18–1.46]<br>*n* = 174	1.23 [1.13–1.38]<br>*n* = 77	1.28 [1.19–1.37]<br>*n* = 25	2.966e-08

Global *p* values were calculated using Kruskal–Wallis tests. Values are median [IQR] and *n*; the IQRs use linear interpolation, whereas the original boxplots use exclusive quartiles. Pairwise NW comparisons use within-marker/sex Bonferroni correction. The cortisol/ACTH ratio uses the units in Section 2.3. BMI category definitions are provided in the Figure 1 legend.

Table S2. Descriptive biomarkers and WHO-compatible model results (continued).

B. WHO-compatible models shown in Figure 5: ratio (95% CI); nominal *p*.

Model	*n*	UW vs NW	OW vs NW	OB vs NW
A: Female ferritin	214	1.036 (0.750–1.431)<br>*p* = 0.8274	0.850 (0.556–1.299)<br>*p* = 0.4498	1.033 (0.567–1.882)<br>*p* = 0.9154
A: Male ferritin	201	0.791 (0.559–1.120)<br>*p* = 0.1851	1.330 (1.042–1.697)<br>*p* = 0.02229	1.461 (0.955–2.234)<br>*p* = 0.08019
B: Cortisol	413	1.224 (1.068–1.403)<br>*p* = 0.003819	0.971 (0.851–1.107)<br>*p* = 0.656	0.686 (0.555–0.846)<br>*p* = 0.0004724
B: ACTH	413	0.941 (0.812–1.090)<br>*p* = 0.4167	1.135 (0.985–1.308)<br>*p* = 0.0801	0.837 (0.667–1.051)<br>*p* = 0.1259
B: Cortisol/ACTH	413	1.301 (1.112–1.522)<br>*p* = 0.001086	0.855 (0.735–0.995)<br>*p* = 0.04254	0.819 (0.643–1.043)<br>*p* = 0.1057
B: FT4	410	1.029 (0.969–1.093)<br>*p* = 0.346	0.947 (0.894–1.003)<br>*p* = 0.06521	1.080 (0.985–1.185)<br>*p* = 0.1029
B: TSH	409	1.219 (0.975–1.524)<br>*p* = 0.08243	1.171 (0.947–1.450)<br>*p* = 0.1449	1.171 (0.832–1.649)<br>*p* = 0.365
B: FT4/TSH	409	0.844 (0.658–1.083)<br>*p* = 0.1829	0.809 (0.637–1.026)<br>*p* = 0.07957	0.922 (0.629–1.351)<br>*p* = 0.6766
B: Ferritin	415	1.001 (0.791–1.267)<br>*p* = 0.9918	1.108 (0.881–1.394)<br>*p* = 0.3786	1.279 (0.886–1.848)<br>*p* = 0.1887

The models use available observations within the 484-person medication-excluded subset of the 572-person WHO-compatible cohort. Figure 5A adjusts for age within each recorded-sex stratum; Figure 5B adjusts for age and recorded sex. Ordinary least-squares models of natural-log biomarkers yield geometric-mean ratios with conventional model-based, pointwise 95% t intervals. N is model-specific; all *p* values are nominal and exploratory. These estimates are distinct from the main-cohort HC3 models in Table S3A and the rank-based comparisons in Section 3.7. BMI category definitions are provided in the Figure 1 legend.

Table S3. Additional exploratory ferritin and recovery models.

A. Male ferritin: ratio (95% CI); nominal/Bonferroni-adjusted *p*.

Adjustment	*n*	UW vs NW	OW vs NW	OB vs NW
Age and lifestyle	313	0.898 (0.686–1.175)<br>0.4319/1	1.219 (1.002–1.483)<br>0.04743/0.1423	1.680 (1.125–2.510)<br>0.01148/0.03443
+ acute severity	313	0.898 (0.685–1.176)<br>0.4324/1	1.217 (0.993–1.491)<br>0.05808/0.1742	1.682 (1.126–2.513)<br>0.01122/0.03365
Adults age and lifestyle	269	0.959 (0.681–1.351)<br>0.8111/1	1.238 (1.007–1.522)<br>0.04269/0.1281	1.686 (1.147–2.477)<br>0.00802/0.02406
Base in ALT/TG sample	298	0.918 (0.701–1.203)<br>0.5362/1	1.193 (0.975–1.459)<br>0.08586/0.2576	1.886 (1.373–2.590)<br>0.0001051/0.0003152
+ log ALT and log TG	298	1.020 (0.797–1.304)<br>0.8765/1	0.982 (0.798–1.207)<br>0.8595/1	1.211 (0.843–1.741)<br>0.2997/0.899

Base models adjust for age per 10 years, past/current smoking, and habitual alcohol use. Ratios have pointwise HC3 t-based 95% CIs; Bonferroni correction covers three BMI contrasts per model. The severity-model base is the same as the 313-person age/lifestyle model and is shown once. Severity compares mild with moderate/severe illness; two ambiguous records are excluded. The ALT/TG models use the same 298 participants and do not establish causal mediation. BMI category definitions are provided in the Figure 1 legend.

B. Pooled recovery models: obesity versus normal weight among men.

Time origin/adjustment	*n*/events	HR (95% CI)	Nominal *p*	Interaction *p*	PH *p* range
Onset: Age-adjusted	565/186	0.390 (0.170–0.894)	0.0261	0.0866	—
Onset: Age-adjusted in lifestyle sample	555/185	0.388 (0.169–0.889)	0.02528	0.08972	—
Onset: Age plus smoking and alcohol	555/185	0.378 (0.165–0.868)	0.02176	0.06122	—
Onset: Adults only, age-adjusted	505/159	0.426 (0.185–0.982)	0.04516	0.1048	—
First visit, age	564/186	0.338 (0.147–0.774)	0.01033	0.0598	Rank 0.353–0.881<br>EA 0.345–0.889
First visit, age + smoking/alcohol	554/185	0.328 (0.143–0.752)	0.008468	0.03964	Rank 0.393–0.888<br>EA 0.386–0.859
Onset with delayed entry, age	564/186	0.304 (0.133–0.697)	0.004915	0.06304	EA 0.280–0.992
Onset with delayed entry, age + smoking/alcohol	554/185	0.302 (0.132–0.693)	0.004733	0.03962	EA 0.148–0.991

All models include obesity, recorded sex, obesity × sex, and age; lifestyle models add smoking/alcohol. Estimates are exploratory, with pointwise CIs and no across-model correction; every model retains six recovery events among men with obesity. HR < 1 indicates a lower recovery rate. A day-zero censored participant is retained in onset-based Cox/KM but excluded from first-visit/delayed-entry Cox; no dates or follow-up times were imputed. First-visit log-rank *p*: men, 0.00883; women, 0.7695. WHO recovery is reported in Section 3.7; no WHO Cox model was fitted.

PH: rank-transformed Schoenfeld test; EA: entry-aware approximate average-event-rank test using Efron risk sets and scaled Schoenfeld residuals. Standard rank testing was not implemented for delayed entry. Ranges cover model terms; onset-model diagnostics are in Section 3.6. No convergence warnings occurred in the four entry-aware models. EA diagnostics do not establish PH or independent truncation.

https://doi.org/10.3390/xxxxx
